# The curious case of APOBEC3 activation by cancer-associated human papillomaviruses

**DOI:** 10.1371/journal.ppat.1006717

**Published:** 2018-01-11

**Authors:** Nicholas A. Wallace, Karl Münger

**Affiliations:** 1 Division of Biology, Kansas State University, Manhattan, Kansas, United States of America; 2 Department of Developmental, Molecular and Chemical Biology, Tufts University School of Medicine, Boston, Massachusetts, United States of America; University of Pittsburgh, UNITED STATES

High-risk human papillomavirus (HR-HPV) infections cause approximately 5% of all human cancers worldwide. These include almost all cervical carcinomas, a leading global cause of cancer deaths, as well as a significant percentage of other anogenital tract cancers and a growing fraction of oropharyngeal carcinomas. To accommodate their life cycles, HR-HPVs need to extensively rewire infected cells. The HR-HPV E6 and E7 proteins are the main drivers of this process, and their expression elicits a barrage of cellular defense responses that restrict this unfriendly takeover of the host cell.

**Fig 1:**
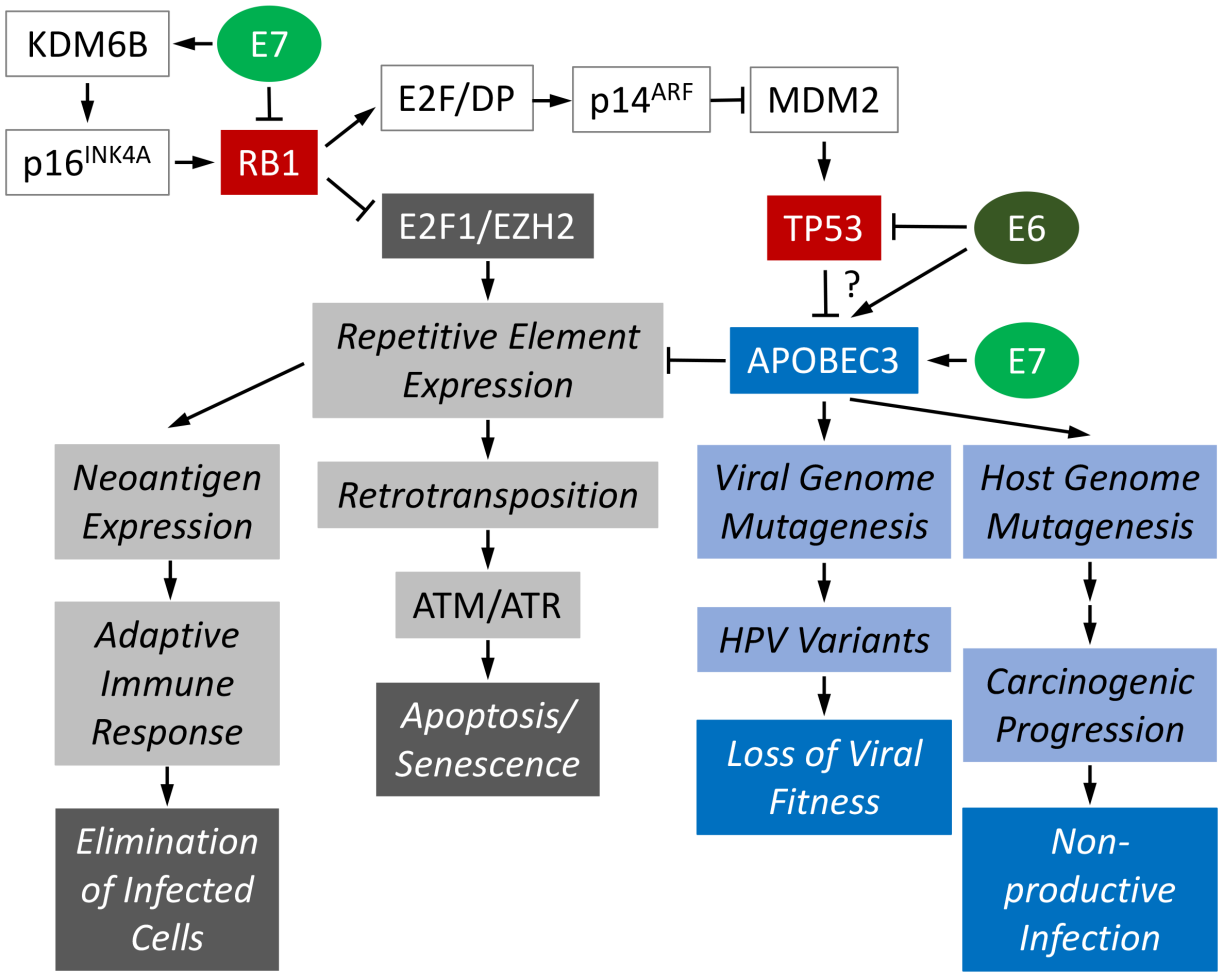
Molecular consequences of APOBEC3 induction in HR-HPV–infected cells. See text for detail. APOBEC3, apolipoprotein B mRNA editing enzyme, catalytic polypeptide-like 3; HR-HPV, high-risk human papillomavirus.

Not surprisingly, HR-HPVs have in turn evolved mechanisms to escape or curb antiviral and anti-oncogenic cellular responses. These mechanisms include degradation of the retinoblastoma (RB1) and the p53 (TP53) tumor suppressors by HR-HPV E7 and E6, respectively. If unopposed, RB1 and TP53 would cause cell cycle arrest, senescence, or cell death in response to HR-HPV infection (Fig 1). Other antiviral pathways, including DNA sensing and interferon signaling, are also blunted by HR-HPV E6 and E7 proteins [1].

Surprisingly, HR-HPVs have not evolved strategies to counteract restriction by apolipoprotein B mRNA editing enzyme, catalytic polypeptide-like 3 (APOBEC3, or simply A3). A3s are interferon-regulated DNA cytosine-to-uracil deaminases encoded as a cluster of seven genes (A3A–A3H; there is no A3E) on human chromosome 22, which are all expressed, albeit at vastly different levels, in epithelial cells [2], which are the natural hosts of HPV infection. While their cytidine deaminase activity causes deoxycytidine (C) to deoxythymidine (T) mutations during viral genome synthesis, A3s also restrict viral replication through cytidine deaminase-independent mechanisms [3].

In response, many viruses have evolved mechanisms to evade A3 restriction. The human immunodeficiency virus 1 (HIV1) Viral infectivity factor (Vif) protein targets A3 family members for degradation, and the HIV2 Viral protein X (Vpx) protein targets A3A for degradation [4, 5]. The Hepatitis B Virus X protein impairs this pathway by packaging A3G into exosomes [6]. Human polyomaviruses—including the Merkel Cell Polyomavirus (McPyV)—trigger A3 activity, yet McPyV-positive Merkel cell carcinomas do not show an A3 mutational signature [7, 8]. This strongly suggests that McPyV overrides A3 restriction [8].

While ectopically expressed HR-HPV E7 and E6 have each been reported to increase expression of A3 family members, and A3A can restrict infection with in vitro–generated HPV16 pseudovirions, A3 activity is not blocked by HR-HPVs [3, 9–11]. Despite the fact that HPV genomes contain fewer than predicted A3 recognition sites [12], the mutational drift caused by A3 mutagenesis is extensive; many of the thousands of HPV16 variants that were detected in a recent study exhibit nucleotide changes that are consistent with A3 action [13]. Furthermore, A3 expression causes nucleotide changes in the host cellular genome. Integration of HPV sequences during malignant progression is accompanied by increased A3A levels [14], and cervical carcinomas and other HPV-associated cancers exhibit A3 mutational signatures [15, 16]. Indeed, A3-mediated host genome destabilization may be one of the mechanisms that drive carcinogenic progression of HR-HPV–associated lesions as evidenced by Phosphatidylinositol-4,5-Bisphosphate 3-Kinase Catalytic Subunit Alpha (PIK3CA) mutations that are consistent with A3 mutagenesis that have been detected in HPV-associated cancers [17]. HPV-associated cancers are generally nonproductive lesions [1], therefore increased carcinogenicity is unlikely to provide an evolutionary benefit to the life cycles of HR-HPVs and does not explain why HR-HPVs have not evolved to block A3 mutagenesis.

Why, then, have HR-HPVs not evolved to subvert this highly active cellular defense response? It has been argued that A3 activation serves to generate viral diversity, and there is no doubt that HPVs are incredibly diverse; the current count stands at more than 300 HPV genotypes, as well as thousands of variants, many of which may have been generated as a result of A3 mutagenesis [13].

The recent discovery by Fred Dick’s research group that RB1 plays a key role in the epigenetic silencing of repetitive elements may provide an alternative explanation as to why it may be beneficial for HR-HPVs not to counteract A3 restriction [18]. RB1 silences repetitive elements by associating with a unique E2F1 transcription factor complex that contains the Enhancer Of Zeste 2 Polycomb Repressive Complex 2 Subunit (EZH2) methyl transferase. The degradation of RB1 by HR-HPV E7 proteins is therefore predicted to cause transcription of repetitive elements [18]. Because translation of some of these—most notably the Long INterspersed Element-1 (LINE1 or L1)—results in neoantigen expression, transcription of repetitive elements would put HR-HPV–infected cells at risk of extinction through adaptive immune responses. Moreover, the LINE1 open reading frame 2 (ORF2) encodes an endonuclease that facilitates the mobilization of LINE1 elements through a “copy and paste” mechanism referred to as retrotransposition. Excessive double-strand DNA breaks caused by the ORF2 endonuclease can result in senescence or apoptosis, which would also lead to the elimination of HR-HPV–infected cells (Fig 1). While the majority of the LINE1 copies in the human genome are 5′ truncated and do not express functional endonucleases, it has been estimated that the remaining functional LINE1 elements may cause 0.3% of all human mutations [19, 20]. This staggering number likely underestimates the mutational impact of LINE1 element mobilization. LINE1 can also mobilize nonautonomous transposable elements, which only requires its second open reading (ORF2) that frequently remains intact even with 5′-truncated LINE1 copies. A3s are well known to restrict expression of repetitive elements, including LINEs, via deaminase-independent activity [21]. Therefore, HR-HPV–infected cells would gain significant advantages from this A3-dependent restriction of LINE elements. Because even unrestrained A3 activity will not completely prevent retrotransposition, the residual activation of double-strand DNA break sensing and repair machinery may be beneficial for efficient HR-HPV genome replication given its well-documented dependence on these factors, including the Ataxia Telangiectasia Mutated (ATM) and Ataxia Telangiectasia And Rad3-Related Protein (ATR) kinases [22].

In addition, HR-HPV–mediated RB1 degradation causes high-level expression of satellite RNAs that can lead to formation of R-loops, a DNA RNA three-stranded structure, which causes replication forks to stall [18]. A3 target the single-stranded DNA in R-loops and can thereby also activate the DNA damage response [23], which benefits HPV replication. Lastly, HR-HPVs may benefit from a deaminase-independent activity of A3, making the A3 mutational signature in HPV-associated tumors simply a by-product of the viruses’ requirement for another function of A3.

Because nearly every unvaccinated, sexually active individual has been infected with an HR-HPV, such an incredibly successful virus should have evolved a defensive strategy against the potent restriction of viral replication and viral persistence by A3 unless it provides them with a selective advantage. HR-HPVs uniquely cause RB1 degradation and thus are predicted to de-repress retrotransposon expression. TP53 is known to restrict retrotransposition [24–26], and HR-HPV E6–mediated TP53 degradation may further increase LINE1 activity. While activation of double-strand DNA sensing and repair pathways induced by retrotransposition triggered by RB1 degradation and R-loop resolution by A3s may stimulate HR-HPV genome replication and progeny synthesis, A3 restriction of repetitive elements may protect HR-HPV–infected cells from undergoing excessive, lethal DNA damage and genomic instability. Moreover, A3 restriction will prevent elimination of HR-HPV–infected cells by adaptive immune responses to neoantigen expression due to expression of repetitive elements.
